# Nut consumption is associated with a shift of the NMR lipoprotein subfraction profile to a less atherogenic pattern among older individuals at high CVD risk

**DOI:** 10.1186/s12933-022-01624-3

**Published:** 2022-09-20

**Authors:** Jesús F. García-Gavilán, Margery A. Connelly, Nancy Babio, Christos S. Matzoros, Emilio Ros, Jordi Salas-Salvadó

**Affiliations:** 1 Universitat Rovira i Virgili, Departament de Bioquímica i Biotecnologia, Unitat de Nutrició Humana, 43201 Reus, Tarragona Spain; 2 Institut d’Investigació Sanitària Pere Virgili (IISPV), Reus, Spain; 3 Consorcio CIBER, Fisiopatología de la Obesidad y Nutrición (CIBERObn), Instituto de Salud Carlos III (ISCIII), Madrid, Spain; 4 Laboratory Corporation of America® Holdings (Labcorp), Morrisville, Raleigh, NC USA; 5 Department of Medicine, Beth Israel Deaconess Medical Center/Harvard Medical School, Boston, MA 02215 USA; 6 Section of Endocrinology, VA Boston Healthcare System, Jamaica Plain, Boston, MA 02130 USA; 7 Lipid Clinic, Department of Endocrinology and Nutrition, Agust Pi i Sunyer Biomedical Research Institute (IDIBAPS), Hospital Clinic, University of Barcelona, Barcelona, Spain

**Keywords:** Advanced lipoprotein, Nuts, Walnuts, Metabolomics

## Abstract

**Background:**

Scientific evidence has accumulated on the beneficial effects of nut consumption on cardiovascular risk and cholesterol reduction, but few studies have examined the effects of nuts on advanced measures of lipoprotein atherogenicity determined by nuclear magnetic resonance (NMR) spectroscopy. We analyzed associations between the amount and type of of nuts consumed and advanced measures of lipoprotein atherogenity and insulin resistance in older individuals at high cardiovascular risk.

**Methods:**

The present observational study was carried out within the framework of the Prevención con Dieta Mediterránea (PREDIMED) trial. Cross-sectional and longitudinal analyses after 1-year of follow-up were conducted in 196 men and women recruited in the PREDIMED-Reus (Spain) center. Dietary intake was assessed using a validated semi-quantitative food questionnaire. Baseline and 1-year fasting plasma lipoprotein and metabolite profiling were performed in plasma using NMR spectra Vantera^®^ Clinical Analyzer. Associations by tertiles of nut consumption between baseline and 1-year changes and advanced measures of lipoprotein atherogenicity, branched chain amminoacids, and measures of insulin resistance were tested by multivariable-adjusted ANCOVA models.

**Results:**

Compared to paticipants in the bottom tertile, those in the top tertile of total nut consumption showed higher levels of large HDL particles and HDL-cholesterol, lower levels of branched-chain amino acids (BCAA) and GlycA, and reduced lipoprotein insulin resistance and diabetes risk index. Participants in the top tertile of walnut consumption disclosed lower levels of very large VLDL, total LDL particles, LDL-cholesterol, and GlycA. Participants in the top tertile of non-walnut nut consumption displayed higher levels of total HDL particles, HDL-cholesterol and apoliporotein A1, lower BCAA and GlycA, and reduced lipoprotein insulin resistance. Participants in the top tertile of 1-year changes in walnut consumption showed increases in medium-sized HDL particles in comparison to the bottom tertile.

**Conclusions:**

In older individuals at high cardiovascular risk, increasing nut consumption was associated with a shift of the NMR lipoprotein subfraction profile to a less atherogenic pattern, as well as lower circulating concentrations of BCAA and decreased insulin resistance. These results provide novel mechanistic insight into the cardiovascular benefit of nut consumption.

*Trial registration* ISRCTN35739639; registration date: 05/10/2005; recruitment start date 01/10/2003.

**Supplementary Information:**

The online version contains supplementary material available at 10.1186/s12933-022-01624-3.

## Background

Nuts are important components of healthy, plant-based dietary patterns [1]. A large body of evidence from prospective cohort studies suggests a beneficial effect of nut consumption on various health outcomes, including reduction of all-cause mortality, cardiovascular disease (CVD), coronary artery disease (CAD), hypertension, atrial fibrillation, and total cancer [2, 3]. The 5-year PREDIMED (PREvención con DIeta MEDiterránea) randomized trial also reported that a Mediterranean diet (MedDiet) supplemented with mixed nuts reduced incident CVD events [4]. Nuts are good sources of healthy nutrients and phytochemicals, such as unsaturated fatty acids, fiber, non-sodium minerals (potassium, calcium, and magnesium), vitamin E, folate, polyphenols, and phytosterols [5], which help explain the positive effects of nuts on the risk of non-communicable diseases and mortality.

Evidence from small, short-term randomized clinical trials in middle-aged individuals indicates a consistent but modest cholesterol-lowering effect of diets supplemented with nuts in general [6] or walnuts in particular [7], which is dose-related and greatest among those with high baseline low-density lipoprotein cholesterol (LDL-C) [8]. Most nut feeding trials have selected young or middle-aged adults [6–8], and few feeding studies have examined the effects of nuts on advanced measures of lipoprotein atherogenicity, such as sub-particle number, size, and composition determined by nuclear magnetic resonance (NMR) spectroscopy. Those who have reported such parameters have shown equivocal results [9].

We hypothesized that nut consumption in general, and walnut consumption in particular, would have a beneficial effect on the lipoprotein profile obtained via NMR. To address this issue, we performed advanced lipoprotein and metabolomic testing in a cohort of older individuals with different levels of total nut and walnut consumption participating in the PREDIMED study, a 5-year nutrition intervention trial testing the effects of a Mediterranean diet supplemented with virgin olive oil or nuts versus a low-fat diet on incident CVD [4].

## Methods

### Study design

The present study was carried out within the framework of the PREDIMED study, a large randomized, multicenter, parallel-group, clinical trial aiming to assess the effects of MedDiet on the primary prevention of CVD in a cohort of older individuals at high cardiovascular risk. Participants were aged between 55 and 80 years and had no CVD at enrollment, but they were at high risk because of the presence of type 2 diabetes or at least three of the following risk factors: current smoking, hypertension, hypercholesterolemia, low high-density lipoprotein (HDL)-cholesterol, overweight or obesity, and family history of premature CVD. Exclusion criteria included any severe chronic illness, drug or alcohol addiction, or allergy or intolerance to olive oil or nuts, two key supplemental foods. In the main study, participants were randomly assigned to three intervention groups: a MedDiet supplemented with virgin olive oil, a MedDiet supplemented with mixed nuts, or a low-fat diet according to the American Heart Association guidelines (control group). The trial is registered at http://www.controlled-trials.com as ISRCTN35739639 and the study protocol and results of the primary outcome have been published elsewhere [4]. The PREDIMED trial was conducted according to the Helsinki Declaration, and the institutional review boards of all recruiting centers approved the study protocol (for the Reus center, the protocol was approved by Hospital Universitari Sant Joan de Reus Ethical Committee). Participants agreed and gave their written informed consent to authorize the use of biological samples for biochemical measurements and genetic studies.

The present cross-sectional and longitudinal analyses were conducted on 196 participants recruited in the PREDIMED-Reus (Spain) center with full food frequency questionnaire (FFQ) data and plasma samples available at baseline and 1 year of follow-up.

### Dietary assessment

Trained dietitians assessed dietary intake in face-to-face interviews at baseline using a validated semi-quantitative 137-item FFQ [10]. For each item, the portion size was established, and nine consumption frequencies were available, ranging from “never or rarely” to “ ≥ 6 times/day”. Energy and nutrient intakes were obtained using data from Spanish food composition tables [11].

Data on self-reported nut consumption were derived from the FFQ, which included an item on the consumption of almonds, peanuts, hazelnuts, pistachios, and pine nuts, and another specific question on the consumption of walnuts. For the present analysis, 28 g of nuts was considered a serving. The number of reported servings was converted into grams per day. The Pearson correlation coefficients for reproducibility and validity of the FFQ regarding nut consumption were 0.66 and 0.38, respectively, and intraclass correlation coefficients for the same measurements in a similar population to the PREDIMED participants were 0.80 and 0.55, respectively [10].

### Covariates

Information about sociodemographic and lifestyle variables, including smoking status, medical conditions, family history of the disease, and medication use, were collected at baseline. Physical activity was estimated with the validated Spanish version of the Minnesota Leisure Time Physical Activity Questionnaire [12]. Trained staff measured height and bodyweight without shoes and wearing light clothing to the nearest 0.5 cm for height and 0.1 kg for bodyweight using a wall-mounted stadiometer and calibrated scales, respectively.

### Lipoprotein and metabolite profiling

Fasting blood samples were collected at baseline and 1-year visits. EDTA plasma was obtained and aliquots were stored at – 80 °C until metabolomic analysis. NMR spectra were acquired on a Vantera^®^ Clinical Analyzer, a 400 MHz NMR instrument, from EDTA plasma samples as described for the NMR LipoProfile^®^ test (Labcorp, Morrisville, NC) [13, 14]. The LP4 deconvolution algorithm was used to report lipoprotein particle concentrations and sizes, as well as concentrations of metabolites such as total branched-chain amino acids, valine, leucine, isoleucine, alanine, glucose, citrate, glycine, total ketone bodies, β-hydroxybutyrate, acetoacetate, acetone [15]. The diameters of the various lipoprotein classes and subclasses are total triglyceride-rich lipoprotein particles (VLDL-P) (24–240 nm), very large VLDL-P (90–240 nm), large VLDL-P (50–89 nm), medium VLDL-P (37–49 nm), small VLDL-P (30–36 nm), very small VLDL-P (24–29 nm), total low-density lipoprotein particles (LDL-P) (19–23 nm), large LDL-P (21.5–23 nm), medium LDL-P (20.5–21.4 nm), small LDL-P (19–20.4 nm), total high-density lipoprotein particles (HDL-P) (7.4–12.0 nm), large HDL-P (10.3–12.0 nm), medium HDL-P (8.7–9.5 nm), and small HDL-P (7.4–7.8 nm). The peak diameters for the largest (H7) to the smallest (H1) of the HDL subspecies are 12.0, 10.8, 10.3, 9.5, 8.7, 7.8, and 7.4 nm. Mean VLDL, LDL, and HDL particle sizes are weighted averages derived from the sum of the diameters of each of the subclasses multiplied by the relative mass percentage. Linear regression against serum lipids measured chemically in a healthy study population (n = 698) provided the conversion factors to generate NMR-derived concentrations of total cholesterol (TC), triglycerides (TG), VLDL-TG, VLDL-C, LDL-C, and HDL-C. NMR-derived concentrations of these parameters are highly correlated with those measured by standard chemistry methods. Details regarding the performance of the assays that quantify branched-chain amino acids (BCAA), citrate, and ketone bodies have been reported [16–18]. Development of the Lipoprotein Insulin Resistance Index (LP-IR) (0–100; least to most insulin resistant), the Diabetes Risk Index (DRI) (1–100; the lowest to the highest risk of type 2 diabetes), and GlycA, a composite measure of systemic inflammation, as well as their analytical and clinical validation, have been published previously [19–21].

### Statistical analyses

Participantsʼ baseline characteristics are described as means ± SD for quantitative traits and percentages for categorical traits. Nut consumption at baseline and 1-year changes were adjusted for total energy intake using the residual method [22]. Nut consumption was categorized into tertiles according to total nuts, walnuts, or non-walnut nuts at baseline and 1-year changes.

Baseline values and 1-year changes in individual lipoprotein, lipid, apolipoprotein, amino acid, ketone bodies, and other molecules were normalized and scaled using Blom's rank-based inverse normal transformation to improve normality [23].

We assessed differences in lipoprotein values between tertiles of nut consumption (total nuts, walnuts, and non-walnut nuts) at baseline using ANCOVA models adjusted by age, gender, body mass index (kg/m^2^), smoking status (ever smoker/never smoker), physical activity (met/day), diabetes (yes/no), dyslipidemia (yes/no), hypertension (yes/no), and statin treatment (yes/no). Data are presented as means and 95% confidence intervals (CI). We also assessed differences in 1-year changes in lipoprotein values between tertiles of nut consumption using ANCOVA models additionally adjusted by the baseline lipid value, baseline nuts consumption, and intervention group (MedDiet + EVOO, MedDiet + Nuts, Low-fat diet). The Tukey test was used to perform multiple comparisons between tertiles. We repeated the same analyses with other molecules including apolipoprotein, amino acids, and ketone bodies.

The assumptions of the ANCOVA models were assessed using visual or quantitative methods. All graphs and tests (Shapiro–Wilk test and Levene’s tests) yielded models that met the criteria for the independence of observations, homogeneity of variance (all Levene’s test *P* values > 0.05), and normality of residuals (all Shapiro–Wilk test *P* values > 0.05).

*P* values < 0.05 were considered statistically significant for these analyses. All statistical analyses were performed with the R software v3.6.1 (www.r-project.org) (R Development Core Team, 2012).

## Results

Participants had a mean age of 67 ± 6 years and 57% were women. The mean BMI was 29.5 ± 3.3 kg/m^2^ and the mean self-reported energy expenditure in physical activity was 263 ± 242 METs/day. 62% of participants never smoked, and the prevalence of T2D was 2%. Mean total nut, walnut and non-walnut consumption at baseline was 14 ± 15 g/day, 7 ± 8 g/day, and 7 ± 9 g/day, respectively.

Table 1 shows the baseline characteristics of the study population by tertiles of energy-adjusted total nut consumption. Mean total nut consumption by tertiles was 3.1 ± 2.9 g/day (tertile 1, T1), 8.5 ± 3.7 g/day (tertile 2, T2), and 29.7 ± 15.3 g/day (tertile 3, T3).

**Table 1:** Baseline participants’ characteristics in a subcohort of the PREDIMED-Reus trial by tertiles of energy-adjusted nut consumption at baseline

	Tertile 1	Tertile 2	Tertile 3	*P*-value
*N*	66	65	65	
Total nuts, g/d	3.1 ± 2.9	8.5 ± 3.7	29.7 ± 15.3	< 0.001
Walnuts, g/d	1.6 ± 2.1	4.3 ± 2.3	13.6 ± 9.4	< 0.001
Non-walnut nuts, g/d	1.5 ± 1.6	4.2 ± 3.3	16.1 ± 11.4	< 0.001
Women, %	52	66	54	0.192
Age, y	66 ± 7	67 ± 6	68 ± 5	0.091
Allocation arm, %				0.154
MedDiet + EVOO	13	10	15	
MedDiet + nuts	11	11	11	
Hypertension, %	97	97	92	0.344
Smoking status, %				0.445
Never	56	68	62	
Current	20	14	15	
Dyslipidemia, %	76	85	85	0.318
Education, %				0.822
≥ Secondary	20	23	15	
Waist circumference, cm	100 ± 9	101 ± 9	100 ± 9	0.801
BMI, kg/m^2^	29.4 ± 3.4	29.9 ± 3.4	29.2 ± 3.3	0.463
Leisure time physical activity, MET-min/day	243.1 ± 238.0	270.0 ± 208.3	277.5 ± 277.3	0.696
Total energy intake, kcal/day	2480 ± 530	2137 ± 477	2426 ± 571	0.001
Glucose, mg/dL	93.0 ± 11.5	90.5 ± 8.9	92.4 ± 12.4	0.403
Triglycerides, mg/dL	138.7 ± 56.2	121.6 ± 53.4	123.8 ± 45.7	0.126
Total cholesterol, mg/dL	224.5 ± 33.6	223.6 ± 36.3	225.9 ± 32.1	0.931
HDL cholesterol, mg/dL	55.7 ± 13.0	60.6 ± 15.4	59.8 ± 12.7	0.097
LDL cholesterol, mg/dL	141.8 ± 28.7	140.6 ± 30.5	142.3 ± 29.3	0.946

Data are means ± SDs unless otherwise stated

**Fig. 1:**
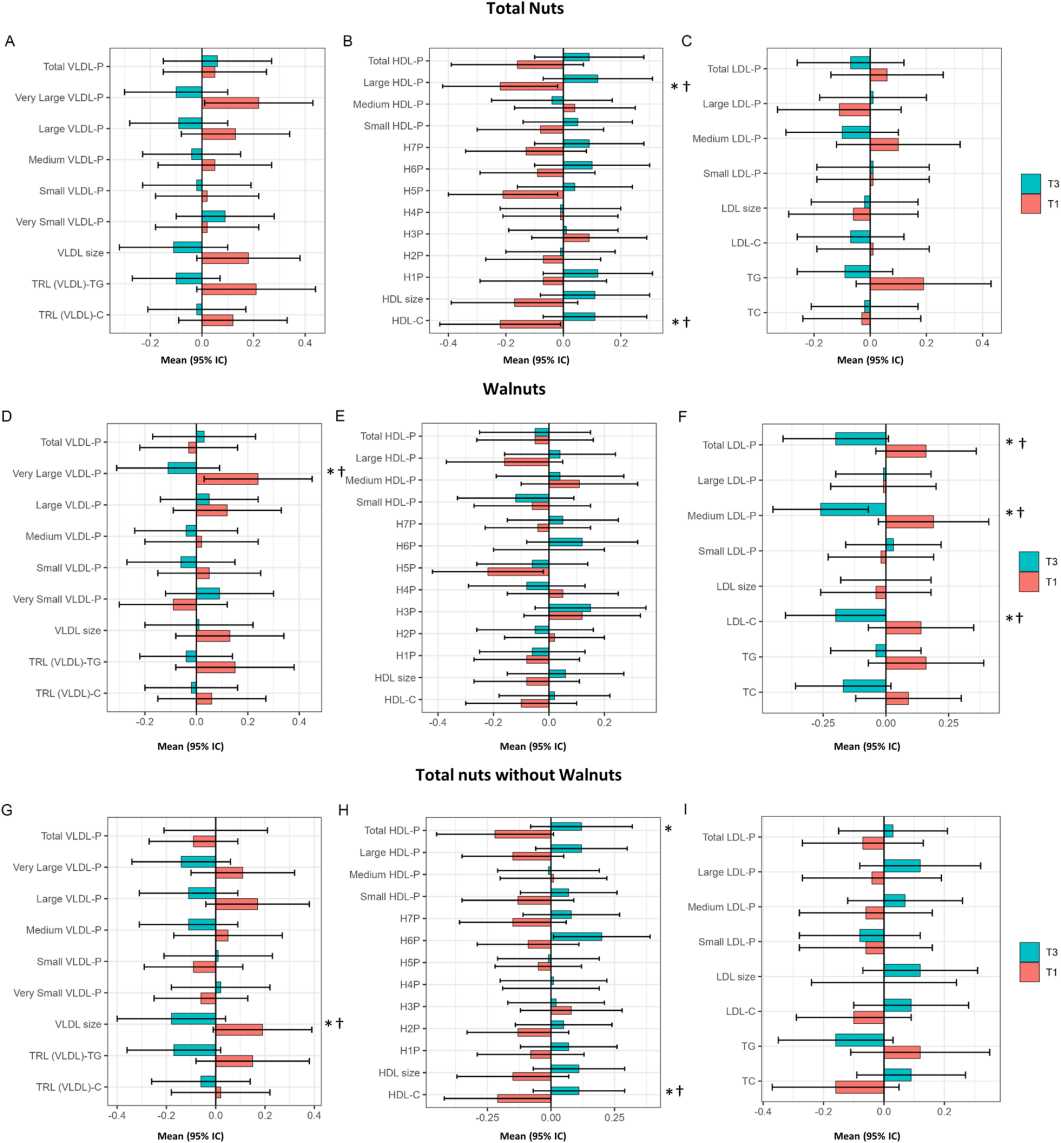
Lipoprotein particle parameters at baseline by tertiles of energy-adjusted nut consumption in a subcohort of the PREDIMED-Reus trial. Metabolomics data are means (95% CI) of normalized values scaled in multiples of 1 SD with Blom’s rank-based inverse normal transformation data. Nuts groups were adjusted by energy intake and values are means (95% CI). The Tukey test was used to perform multiple comparisons between tertiles. *P-value < 0.05 in ANCOVA adjusted by age, gender, body mass index (kg/m2), smoking status (ever smoker/never smoker), physical activity (met/day), diabetes (yes/no), dyslipidemia (yes/no), hypertension (yes/no), and statin treatment (yes/no). P-value < 0.05 for comparison between T1 and T3 after the Tukey test. LDL, low-density lipoprotein; HDL, high-density lipoprotein; TRL-P, Triglyceride-Rich Lipoprotein Particle; TG, triglyceride; TC, total cholesterol; TRL, Triglyceride-Rich Lipoprotein; VLDL, very low-density lipoprotein; T1, tertile 1; T3, tertile 3

Figure 1 and Additional file 1: Table S1 shows the differences between baseline tertiles of energy-adjusted nut consumption by nut subtypes (total nuts, walnuts, and non-walnut nuts) and normalized values of lipoprotein particles at baseline. Significant differences in baseline values between tertiles of nut consumption were observed for some lipoprotein particles. Large HDL-P (total nuts) and HDL-C (total nuts and non-walnut nuts) were higher in participants in the top tertile compared to those in the lower tertile, whereas TRL (VLDL) size was lower in participants with higher consumption of non-walnut nuts (Fig. 1G, H, I). Compared to participants in the lowest tertile of walnut consumption, very large VLDL-P, total LDL-P, medium LDL-P, and LDL-C were lower in those in the top tertile (Fig. 1D, E, F).

Table 2 shows the differences between tertiles of 1-year changes in energy-adjusted nut consumption and 1-year changes in normalized values of lipoprotein particles. Participants in the top tertile of 1-year changes in walnut consumption showed a higher increase in H7P and H4P. In addition, non-significant increases in very large VLDL-P (*P*-value = 0.057 for total nuts and *P*-value = 0.076 for non-walnuts nuts), and H3P (*P*-value = 0.083 for walnuts) were observed in participants in the top tertile of 1-year changes in these food groups.

**Table 2:** 1-year changes of lipoprotein particle parameters by tertiles of 1-year energy-adjusted nut consumption in a subcohort of the PREDIMED-Reus trial

Variables	Tertile 1 (n = 65)	Tertile 3 (n = 65)	ANCOVA *P*-value	Tertile 1 (n = 65)	Tertile 3 (n = 65)	ANCOVA *P*-value	Tertile 1 (n = 65)	Tertile 3 (n = 65)	ANCOVA *P*-value
	Total nuts			Walnuts			Non-walnut nuts		
Food, g/d	− 21.90 (− 24.01, − 19.79)	26.41 (23.88, 28.94)		− 11.85 (− 12.96,− 10.74)	14.21 (12.93, 15.49)		− 12.37 (− 14.00,− 10.74)	14.73 (13.45, 16.01)	
Triglyceride-rich lipoprotein (VLDL) particle concentrations									
Total	− 0.01 (− 0.22, 0.20)	− 0.02 (− 0.21, 0.17)	0.938	− 0.07 (− 0.28, 0.14)	0.04 (− 0.14, 0.22)	0.735	− 0.01 (− 0.23, 0.21)	0.00 (− 0.19, 0.19)	0.974
Very large	− 0.24 (− 0.46, − 0.02)	0.07 (− 0.19, 0.33)	0.057	− 0.01 (− 0.24, 0.22)	0.04 (− 0.20, 0.28)	0.790	− 0.24 (− 0.47, − 0.01)	0.05 (− 0.21, 0.31)	0.079
Large	0.00 (− 0.21, 0.21)	0.00 (− 0.20, 0.20)	0.983	− 0.01 (− 0.20, 0.18)	0.04 (− 0.17, 0.25)	0.842	− 0.02 (− 0.23, 0.19)	0.05 (− 0.16, 0.26)	0.636
Medium	0.10 (− 0.11, 0.31)	− 0.08 (− 0.28, 0.12)	0.235	0.06 (− 0.14, 0.26)	0.00 (− 0.21, 0.21)	0.805	0.08 (− 0.13, 0.29)	0.03 (− 0.18, 0.24)	0.719
Small	− 0.06 (− 0.29, 0.17)	− 0.08 (− 0.30, 0.14)	0.925	− 0.06 (− 0.28, 0.16)	− 0.08 (− 0.30, 0.14)	0.338	− 0.05 (− 0.28, 0.18)	− 0.16 (− 0.38, 0.06)	0.461
Very small	0.04 (− 0.21, 0.29)	0.05 (− 0.18, 0.28)	0.963	− 0.01 (− 0.25, 0.23)	0.10 (− 0.14, 0.34)	0.514	− 0.01 (− 0.26, 0.24)	0.07 (− 0.16, 0.30)	0.626
LDL particle concentrations									
Total	0.00 (− 0.15, 0.15)	0.00 (− 0.19, 0.19)	0.998	0.03 (− 0.12, 0.18)	0.01 (− 0.18, 0.20)	0.798	0.01 (− 0.13, 0.15)	− 0.09 (− 0.28, 0.10)	0.472
Large	− 0.10 (− 0.24, 0.04)	0.05 (− 0.10, 0.20)	0.253	− 0.13 (− 0.29, 0.03)	0.00 (− 0.15, 0.15)	0.143	0.02 (− 0.13, 0.17)	− 0.07 (− 0.24, 0.10)	0.490
Medium	0.09 (− 0.18, 0.36)	− 0.04 (− 0.28, 0.20)	0.427	0.05 (− 0.23, 0.33)	− 0.04 (− 0.26, 0.18)	0.800	− 0.03 (− 0.31, 0.25)	− 0.02 (− 0.27, 0.23)	0.957
Small	0.01 (− 0.19, 0.21)	0.02 (− 0.15, 0.19)	0.939	0.04 (− 0.15, 0.23)	0.06 (− 0.12, 0.24)	0.507	0.01 (− 0.20, 0.22)	− 0.02 (− 0.19, 0.15)	0.838
HDL particle concentrations									
Total	− 0.07 (− 0.23, 0.09)	0.12 (− 0.04, 0.28)	0.139	− 0.09 (− 0.24, 0.06)	0.06 (− 0.10, 0.22)	0.406	− 0.15 (− 0.32, 0.02)	0.07 (− 0.09, 0.23)	0.079
Large	0.07 (− 0.07, 0.21)	0.01 (− 0.09, 0.11)	0.517	0.05 (− 0.08, 0.18)	0.00 (− 0.10, 0.10)	0.729	0.10 (− 0.05, 0.25)	− 0.01 (− 0.11, 0.09)	0.302
Medium	− 0.08 (− 0.23, 0.07)	0.00 (− 0.17, 0.17)	0.529	− 0.18 (− 0.35, − 0.01)	0.00 (− 0.17, 0.17)	0.038	− 0.10 (− 0.26, 0.06)	− 0.04 (− 0.22, 0.14)	0.642
Small	− 0.05 (− 0.22, 0.12)	0.09 (− 0.06, 0.24)	0.243	− 0.05 (− 0.20, 0.10)	0.06 (− 0.09, 0.21)	0.666	− 0.09 (− 0.27, 0.09)	0.09 (− 0.07, 0.25)	0.138
H7P	0.02 (− 0.16, 0.20)	0.18 (0.00, 0.36)	0.270	− 0.26 (− 0.45, − 0.07)	0.18 (0.00, 0.36)	0.005	0.01 (− 0.18, 0.20)	0.19 (0.00, 0.38)	0.214
H6P	− 0.04 (− 0.24, 0.16)	− 0.04 (− 0.24, 0.16)	0.997	0.03 (− 0.17, 0.23)	− 0.06 (− 0.26, 0.14)	0.838	− 0.02 (− 0.23, 0.19)	− 0.13 (− 0.33, 0.07)	0.465
H5P	0.09 (− 0.12, 0.30)	0.06 (− 0.12, 0.24)	0.838	0.11 (− 0.09, 0.31)	0.11 (− 0.07, 0.29)	0.072	0.15 (− 0.07, 0.37)	0.11 (− 0.09, 0.31)	0.793
H4P	− 0.03 (− 0.25, 0.19)	− 0.05 (− 0.24, 0.14)	0.853	− 0.19 (− 0.41, 0.03)	− 0.07 (− 0.27, 0.13)	0.045	− 0.06 (− 0.28, 0.16)	− 0.10 (− 0.29, 0.09)	0.796
H3P	− 0.10 (− 0.31, 0.11)	0.08 (− 0.11, 0.27)	0.209	− 0.13 (− 0.32, 0.06)	0.12 (− 0.06, 0.30)	0.240	− 0.13 (− 0.35, 0.09)	0.06 (− 0.13, 0.25)	0.214
H2P	− 0.09 (− 0.26, 0.08)	− 0.01 (− 0.18, 0.16)	0.592	− 0.09 (− 0.25, 0.07)	0.01 (− 0.16, 0.18)	0.599	− 0.12 (− 0.30, 0.06)	0.01 (− 0.16, 0.18)	0.332
H1P	− 0.01 (− 0.17, 0.15)	0.14 (− 0.02, 0.30)	0.249	0.02 (− 0.14, 0.18)	0.06 (− 0.10, 0.22)	0.626	− 0.01 (− 0.17, 0.15)	0.13 (− 0.03, 0.29)	0.255
Mean lipoprotein sizes: particle size									
VLDL	− 0.05 (− 0.27, 0.17)	0.06 (− 0.11, 0.23)	0.427	0.01 (− 0.18, 0.20)	0.04 (− 0.15, 0.23)	0.947	− 0.07 (− 0.30, 0.16)	0.08 (− 0.10, 0.26)	0.308
LDL	− 0.08 (− 0.24, 0.08)	0.03 (− 0.12, 0.18)	0.373	− 0.14 (− 0.30, 0.02)	0.06 (− 0.10, 0.22)	0.125	0.00 (-0.16, 0.16)	-0.04 (-0.19, 0.11)	0.747
HDL	0.01 (-0.10, 0.12)	0.03 (-0.06, 0.12)	0.879	-0.05 (-0.17, 0.07)	0.01 (-0.09, 0.11)	0.585	0.05 (-0.07, 0.17)	0.00 (-0.11, 0.11)	0.574
Derived triglyceride and cholesterol (concentrations)									
TG	− 0.02 (− 0.21, 0.17)	0.01 (− 0.16, 0.18)	0.796	0.01 (− 0.16, 0.18)	0.04 (− 0.13, 0.21)	0.829	− 0.02 (− 0.21, 0.17)	0.03 (− 0.14, 0.20)	0.687
TC	− 0.04 (− 0.21, 0.13)	0.04 (− 0.15, 0.23)	0.597	− 0.06 (− 0.23, 0.11)	0.04 (− 0.16, 0.24)	0.780	− 0.01 (− 0.18, 0.16)	− 0.07 (− 0.26, 0.12)	0.681
TRL (VLDL)-TG	− 0.02 (− 0.21, 0.17)	0.01 (− 0.16, 0.18)	0.868	0.01 (− 0.17, 0.19)	0.05 (− 0.11, 0.21)	0.776	− 0.02 (− 0.21, 0.17)	0.05 (− 0.12, 0.22)	0.591
TRL (VLDL)-C	− 0.02 (− 0.21, 0.17)	0.01 (− 0.17, 0.19)	0.872	− 0.06 (− 0.24, 0.12)	0.06 (− 0.11, 0.23)	0.647	− 0.02 (− 0.21, 0.17)	0.03 (− 0.15, 0.21)	0.738
LDL-C	− 0.04 (− 0.20, 0.12)	0.00 (− 0.19, 0.19)	0.752	− 0.01 (− 0.17, 0.15)	0.02 (− 0.17, 0.21)	0.961	0.00 (− 0.14, 0.14)	− 0.11 (− 0.30, 0.08)	0.411
HDL-C	0.00 (− 0.11, 0.11)	0.05 (− 0.06, 0.16)	0.594	− 0.10 (− 0.22, 0.02)	0.03 (− 0.07, 0.13)	0.221	0.00 (− 0.11, 0.11)	0.00 (− 0.11, 0.11)	0.942

Lipid data are means (95% CI) of normalized values scaled in multiples of 1 SD with Blom’s rank-based inverse normal transformation data. Nuts groups were adjusted by energy intake

*P*-values were obtained by ANCOVA adjusted by age, gender, body mass index (kg/m^2^), smoking status (ever smoker/never smoker), physical activity (met/day), diabetes (yes/no), dyslipidemia (yes/no), hypertension (yes/no), statin treatment (yes/no), baseline normalized lipidomic values and baseline nut consumption

*LDL* low-density lipoprotein, *HDL* high-density lipoprotein, *TRL-P* Triglyceride-Rich Lipoprotein Particle, *TG* triglyceride, *TC* total cholesterol, *TRL* Triglyceride-Rich Lipoprotein, *VLDL* very low-density lipoprotein

^a^*P*-value < 0.05 for comparison between T1 and T3 after the Tukey test

**Fig. 2:**
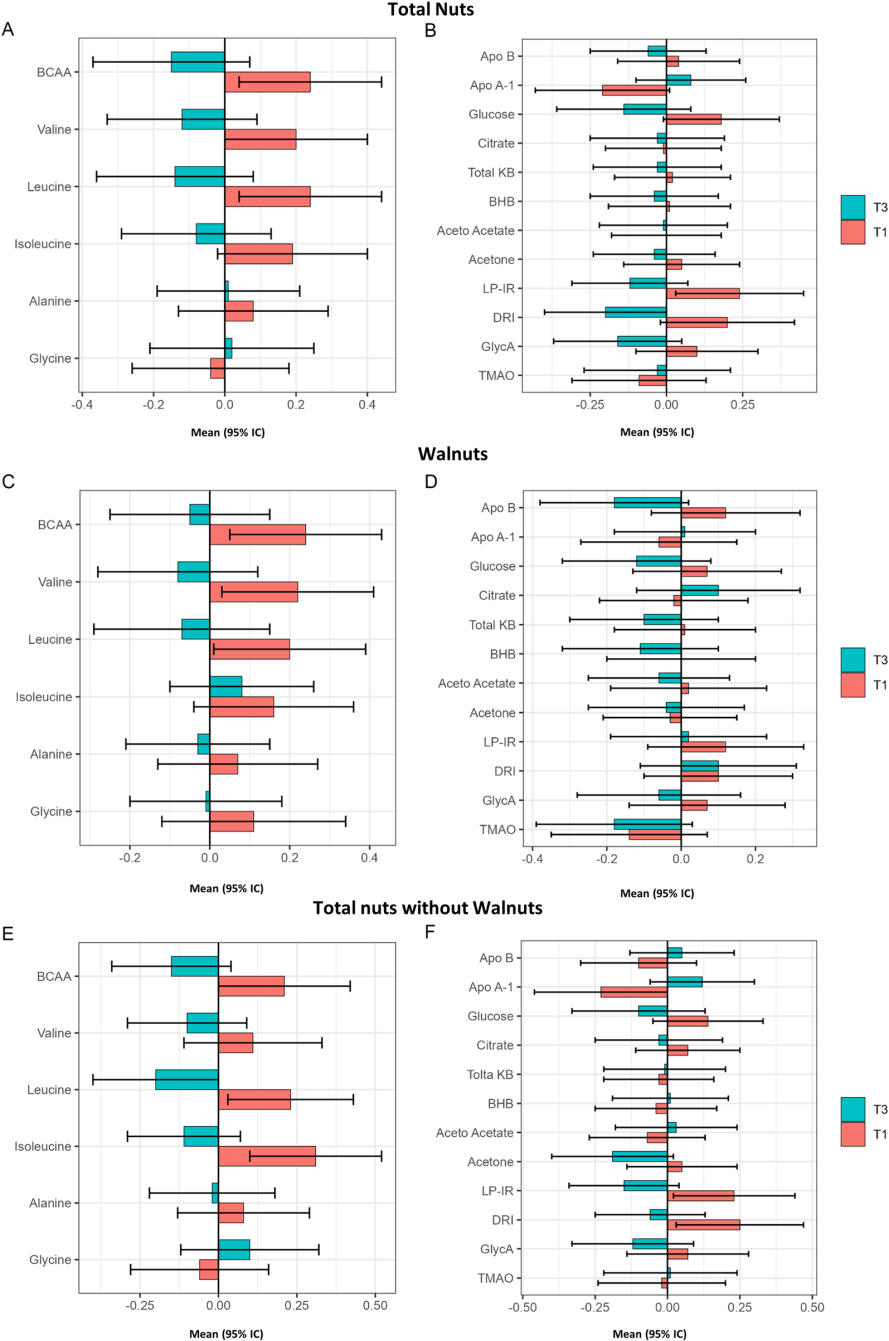
Apolipoproteins, small molecule metabolites, and markers of diabetes risk at baseline by tertiles of baseline energy-adjusted nut consumption in a subcohort of the PREDIMED-Reus trial. Metabolomics data are means (95% CI) of normalized values scaled in multiples of 1 SD with Blom’s rank-based inverse normal transformation data. Nuts groups were adjusted by energy intake and values are means (95% CI). The Tukey test was used to perform multiple comparisons between tertiles. *P*-value < 0.05 in ANCOVA adjusted by age, gender, body mass index (kg/m2), smoking status (ever smoker/never smoker), physical activity (met/day), diabetes (yes/no), dyslipidemia (yes/no), hypertension (yes/no), and statin treatment (yes/no). *P*-value < 0.05 for comparison between T1 and T3 after the Tukey test. Apo, apolipoprotein; BCAA, Branched-Chain Amino Acids; KB, ketone body; LP-IR, lipoprotein insulin resistance; DRI, diabetes risk index; TMAO, Trimethylamine N-oxide; T1, tertile 1; T3, tertile 3

Differences between the tertiles of total nuts, walnuts, or non-walnut nuts consumption and other metabolites at baseline are shown in Fig. 2 and Additional file 1: Table S2. ApoA1 was higher in top consumers of non-walnut nuts. Participants in the highest tertile of total nut consumption (Fig. 2A) showed lower concentrations of BCAA, valine, and leucine; those with higher consumption of walnuts (Fig. 2C) had lower concentrations of BCAA and valine; and participants in the top tertile of non-walnut nut consumption (Fig. 2E) exhibited lower concentrations of BCAA, leucine, and isoleucine. There were no between-group differences at baseline in glucose, citrate, or ketone bodies. Concerning diabetes risk markers, GlycA was lower in top nut consumers of all groups (Fig. 2B, D, F), while LP-IR was lower in top consumers of total nuts (Fig. 2B) and non-walnut nuts (Fig. 2F), and DRI was lower only in top consumers of total nuts (Fig. 2B).

Table 3 shows the differences between tertiles of 1-year changes in the different types of energy-adjusted nuts and 1-year changes in normalized values of other molecules. Participants in the top tertile of 1-year changes in walnut consumption showed a larger decrease in plasma glycine levels in comparison to the lower tertile (*p*-value = 0.030).

**Table 3:** 1-year changes of apolipoproteins, small molecule metabolites, and markers of diabetes risk by tertiles of 1-year changes of energy-adjusted nuts consumption in a subcohort from PREDIMED-Reus trial

Variables	Tertile 1 (n = 65)	Tertile 3 (n = 65)	ANCOVA *P*-value	Tertile 1 (n = 65)	Tertile 3 (n = 65)	ANCOVA *P*-value	Tertile 1 (n = 65)	Tertile 3 (n = 65)	ANCOVA *P*-value
	Total nuts			Walnuts			Non-walnut nuts		
Food, g/d	− 21.90 (− 24.01, − 19.79)	26.41 (23.88, 28.94)		− 11.85 (− 12.96, − 10.74)	14.21 (12.93, 15.49)		− 12.37 (− 14.00, − 10.74)	14.73 (13.45, 16.01)	
Apolipoprotein concentrations									
Apo B	− 0.02 (− 0.19, 0.15)	− 0.01 (− 0.19, 0.17)	0.913	− 0.02 (− 0.19, 0.15)	0.02 (− 0.16, 0.20)	0.948	− 0.01 (− 0.17, 0.15)	− 0.10 (− 0.28, 0.08)	0.514
Apo A-1	− 0.04 (− 0.17, 0.09)	0.03 (− 0.11, 0.17)	0.531	− 0.07 (− 0.20, 0.06)	0.01 (− 0.13, 0.15)	0.473	− 0.09 (− 0.22, 0.04)	− 0.03 (− 0.17, 0.11)	0.577
Amino acid concentrations									
BCAA	0.03 (− 0.12, 0.18)	0.13 (− 0.03, 0.29)	0.452	− 0.01 (− 0.17, 0.15)	0.10 (− 0.06, 0.26)	0.562	0.07 (− 0.08, 0.22)	0.02 (− 0.14, 0.18)	0.708
Valine	0.04 (− 0.13, 0.21)	0.03 (− 0.15, 0.21)	0.946	0.03 (− 0.14, 0.20)	0.05 (− 0.13, 0.23)	0.902	0.04 (− 0.13, 0.21)	− 0.01 (− 0.20, 0.18)	0.743
Leucine	0.00 (− 0.18, 0.18)	0.22 (0.05, 0.39)	0.106	− 0.03 (− 0.22, 0.16)	0.15 (− 0.02, 0.32)	0.269	0.08 (− 0.09, 0.25)	0.03 (− 0.15, 0.21)	0.741
Isoleucine	0.07 (− 0.11, 0.25)	0.09 (− 0.15, 0.33)	0.870	− 0.07 (− 0.24, 0.10)	0.11 (− 0.12, 0.34)	0.485	0.11 (− 0.07, 0.29)	0.09 (− 0.13, 0.31)	0.877
Alanine	0.04 (− 0.16, 0.24)	0.09 (− 0.09, 0.27)	0.751	0.04 (− 0.16, 0.24)	0.00 (− 0.19, 0.19)	0.888	0.06 (− 0.15, 0.27)	0.04 (− 0.14, 0.22)	0.883
Glycine	0.09 (− 0.03, 0.21)	− 0.04 (− 0.18, 0.10)	0.213	0.15 (0.02, 0.28)	− 0.09 (− 0.24, 0.06)^a^	0.066	0.08 (− 0.04, 0.20)	0.01 (− 0.15, 0.17)	0.506
Small molecule metabolites									
Glucose	− 0.07 (− 0.24, 0.10)	0.06 (− 0.11, 0.23)	0.311	− 0.06 (− 0.23, 0.11)	− 0.02 (− 0.20, 0.16)	0.563	− 0.09 (− 0.27, 0.09)	0.06 (− 0.11, 0.23)	0.253
Citrate	0.04 (− 0.20, 0.28)	0.00 (− 0.21, 0.21)	0.813	− 0.07 (− 0.30, 0.16)	0.06 (− 0.16, 0.28)	0.713	0.01 (− 0.24, 0.26)	0.06 (− 0.15, 0.27)	0.729
Ketone body concentrations									
Total KB	0.01 (− 0.28, 0.30)	− 0.13 (− 0.34, 0.08)	0.429	− 0.09 (− 0.38, 0.20)	0.01 (− 0.21, 0.23)	0.474	0.00 (− 0.31, 0.31)	− 0.10 (− 0.32, 0.12)	0.576
Beta-hydroxy-butyrate	0.07 (− 0.20, 0.34)	− 0.21 (− 0.43, 0.01)	0.098	− 0.01 (− 0.29, 0.27)	− 0.03 (− 0.26, 0.20)	0.843	0.03 (− 0.27, 0.33)	− 0.14 (− 0.37, 0.09)	0.311
Aceto-acetate	− 0.08 (− 0.38, 0.22)	0.02 (− 0.23, 0.27)	0.568	− 0.17 (− 0.47, 0.13)	0.04 (− 0.21, 0.29)	0.125	− 0.11 (− 0.40, 0.18)	− 0.06 (− 0.31, 0.19)	0.757
Acetone	0.07 (− 0.23, 0.37)	− 0.09 (− 0.36, 0.18)	0.355	0.03 (− 0.28, 0.34)	0.06 (− 0.20, 0.32)	0.812	0.01 (− 0.28, 0.30)	− 0.01 (− 0.27, 0.25)	0.910
Diabetes risk multimarkers									
LP-IR	0.02 (− 0.14, 0.18)	− 0.01 (− 0.16, 0.14)	0.816	0.05 (− 0.11, 0.21)	0.04 (− 0.11, 0.19)	0.455	0.02 (− 0.14, 0.18)	0.01 (− 0.16, 0.18)	0.942
DRI	0.05 (− 0.19, 0.29)	0.14 (− 0.13, 0.41)	0.603	0.01 (− 0.22, 0.24)	0.13 (− 0.15, 0.41)	0.407	0.02 (− 0.24, 0.28)	0.08 (− 0.18, 0.34)	0.705
GlycA	0.05 (− 0.13, 0.23)	− 0.15 (− 0.30, 0.00)	0.096	0.00 (− 0.18, 0.18)	− 0.16 (− 0.31, − 0.01)	0.148	0.00 (− 0.19, 0.19)	− 0.12 (− 0.25, 0.01)	0.309
TMAO	0.18 (− 0.07, 0.43)	− 0.10 (− 0.33, 0.13)	0.098	0.08 (− 0.20, 0.36)	− 0.21 (− 0.44, 0.02)	0.103	0.10 (− 0.16, 0.36)	− 0.12 (− 0.35, 0.11)	0.214

Metabolic data are means (95% CI) of normalized values scaled in multiples of 1 SD with Blom’s rank-based inverse normal transformation data. 1-year changes in nuts consumption values are means ± SD. *P*-values were obtained by ANCOVA adjusted by age, gender, body mass index (kg/m^2^), smoking status (ever smoker/never smoker), physical activity (met/day), diabetes (yes/no), dyslipidemia (yes/no), hypertension (yes/no), and statin treatment (yes/no).

1-year change ANCOVAs were additionally adjusted by baseline metabolic values and baseline nut consumption

*Apo* apolipoprotein, *BCAA* Branched-Chain Amino Acids, *KB*, ketone body, *LP-IR* lipoprotein insulin resistance, *DRI* diabetes risk index, *TMAO* Trimethylamine N-oxide

^a^*P*-value < 0.05 for comparison between T1 and T3 after the Tukey test

## Discussion

The results of this cross-sectional and longitudinal analysis of 196 participants in the PREDIMED study, a feeding trial in which the diets were supplemented or not with nuts, support an antiatherogenic effect of nut consumption on lipoprotein subfractions as assessed by NMR. At baseline, increasing consumption of total nuts, walnuts, and non-walnut nuts was associated with increased HDL-C; decreased total and medium LDL-P, very large VLDL, and LDL-C; and decreased VLDL particle size and increased HDL-P and HDL-C, respectively, in multivariable models that included adjustment for non-lipid cardiovascular risk factors and statin use. In the longitudinal study, HDL sub-particles H7P and H4P increased in the upper category of walnut consumption.

### High-density lipoproteins

First, higher consumption of total nuts and non-walnut nuts was associated with increased HDL-C. This finding is counter to known evidence on the null effect of nuts on HDL-C, as summarized in meta-analyses [6–8]. As large HDL-P contain more cholesterol, their increase with higher total nut consumption supports higher HDL-C. Both total HDL-P and ApoA1 were also higher in the top category of non-walnut nut consumption. Of note, HDL-P was more strongly associated with measures of CAD and was a better predictor of incident CVD events than HDL-C in high-risk statin-treated patients [24], as well as in a pooled analysis of cohorts free of CVD [25]. Interestingly, increased walnut consumption in the longitudinal analysis was also associated with increased H4P and H7P HDL-P. H4P are medium HDL subspecies that have been inversely associated with the development of type-2 diabetes in a large population-based cohort study [26], while H7P are the largest HDL-P and carry the highest cholesteryl ester content.

### Triglyceride-rich lipoproteins

Second, increased consumption of walnuts and non-walnut nuts was associated with a reduction of very large VLDL particles and VLDL size, respectively. While large VLDL were initially considered pro-atherogenic, discordant results have been obtained in recent studies and presently it is the small VLDL, which make up remnants, that is considered the most atherogenic TG-rich lipoprotein species [27]. Thus, the present findings on NMR-related VLDL characteristics are not easily interpretable in terms of CVD risk. However, these findings do suggest a reduction in diabetic dyslipidemia, which is prevalent in metabolic disease and insulin resistance [28–30].

### Low-density lipoproteins

Third, increasing dietary walnuts was associated with reduced LDL-C and LDL-P. That nuts in general and walnuts, in particular, have an LDL-C lowering effect has been consistently observed in feeding trials [6–8]. Lower LDL-P with increasing walnut doses is an important finding. As reviewed [31], in the last 15 years large prospective studies focusing on CVD outcomes have reported that LDL-P consistently outperforms LDL-C in CVD risk prediction, which is due to the fact that some individuals, particularly those with increased triglycerides, disclose increased LDL-P numbers while having normal LDL-C. In our study, nut consumption was unrelated to the concentrations of small LDL-P and LDL subspecies that are closely related to TG-rich lipoproteins and with CVD risk independently of LDL-C [31].

### Comparison with other studies

Lipoprotein subclass phenotyping by NMR or other techniques has been performed in a few nut-feeding studies. A recent review [9] summarized the results of 5 controlled nut trials that reported data on small LDL, which decreased in 3 of them. Three controlled nut trials have reported LDL-P changes by NMR: LDL-P was reduced with mixed nuts with a predominance of walnuts in one study [32], while pistachios had no effect in another trial [33]. Recently, a large 2-year randomized trial has reported again a significant reduction of both total and small LDL-P with a diet containing walnuts at 15% of energy compared with a control diet [34]. The present findings add evidence to the antiatherogenic shift of LDL particles with nuts, particularly walnuts.

### Advanced metabolomic analyses

Concerning other advanced metabolomic analyses, total circulating BCAA and concentrations of valine, leucine, and isoleucine were variably lower at baseline in the top categories of nut consumption. Plasma BCAA have been related to the risk of both CVD [20, 35] and diabetes [36]. Case-cohort studies within the PREDIMED study have also uncovered associations of baseline circulating BCAA with CVD [37] and diabetes [38]. Of note, the Mediterranean diet enriched with nuts appeared to offset the CVD risk associated with increased BCAAs [37]. Measures of insulin resistance (LP-IR) and risk of incident diabetes (DRI) were also lower with higher baseline nut consumption. This is consistent with the reduced very large VLDL particles and increased large HDL particles and HDL-C. This is also in line with limited research that has evaluated the long-term effect of nut consumption on glycemic markers. A pooled analysis of intervention trials conducted in individuals with or without diabetes concluded that nut consumption reduced fasting insulin and HOMA-IR, whereas no effects on glycated hemoglobin (HbA1c) or fasting glucose were observed [39]. However, increasing nut consumption in the longitudinal study did not affect future risk of insulin resistance or diabetes. Finally, baseline GlycA, a novel biomarker of systemic inflammation and cardiometabolic risk [21, 40], was consistently reduced with increased nut consumption. Given that increased GlycA levels are associated with future CVD events and diabetes, these results further suggest that nut consumption has beneficial effects on cardiometabolic risk.

### Mechanistic insights

The association of nut consumption with antiatherogenic shifts of NMR lipoproteins and improvement of both the blood amino acid profile, insulin resistance, and GlycA shown in this study contributes novel mechanistic insight into the known benefit of nut consumption on CVD risk [2–5]. Nuts are rich in unsaturated fats, soluble fiber, polyphenols, and phytosterols [5], which help explain their beneficial effect on lipid metabolism.

### Limitations and strenghts

Our study has limitations. While at baseline all participants ate nuts on their own, during follow-up those participating in one of the trial arms were given nuts for free, which biases consumption in one subgroup. Additionally, the study subjects were older individuals at high CVD risk, thus the results cannot be generalized to younger individuals. There are also strengths to our study, such as the measurement of lipoprotein subfractions with an up-to-date NMR spectroscopy technique, which provides precise physical–chemical data on a wide range of lipoprotein particles.

## Conclusions

In conclusion, in older individuals at high CVD risk, increasing nut consumption was associated with a shift of the NMR lipoprotein subfraction profile to a less atherogenic pattern, as well as lower circulating concentrations of BCAA and reduced insulin resistance and diabetes risk index, two specific NMR measures. The present results provide novel mechanistic insight into the benefit of nut consumption on CVD risk.

## Supplementary Information

**Additional file 1: Table S1.** Lipoprotein particle parameters at baseline by tertiles of energy-adjusted nut consumption in a subcohort of the PREDIMED-Reus trial. **Table S2.** Apolipoproteins, small molecule metabolites, and markers of diabetes risk at baseline by tertiles of baseline energy-adjusted nut consumption at baseline in a subcohort of the PREDIMED-Reus trial.

## Data Availability

The datasets generated and/or analyzed during the current study are not publicly available due to the lack of authorization from PREDIMED participants but are available from the corresponding author on reasonable request.

## Abbreviations

BCAA: Branched-chain amino acids
CAD: Coronary artery disease
CVD: Cardiovascular disease
DRI: The diabetes risk index
FFQ: Food frequency questionnaire
HDL: High-density lipoprotein
HDL-P: High-density lipoprotein particles
LDL-C: Low-density lipoprotein cholesterol
LDL-P: Low-density lipoprotein particles
LP-IR: The lipoprotein insulin resistance index
MedDiet: Mediterranean diet
NMR: Nuclear magnetic resonance spectroscopy
PREDIMED: PREvención con DIeta MEDiterránea
T1: Tertile 1
T2: Tertile 2
T3: Tertile 3
TC: Total cholesterol
TG: Triglycerides
TRL-P: Triglyceride-rich lipoprotein particles
