# Correction: Nut consumption is associated with a shift of the NMR lipoprotein subfraction profile to a less atherogenic pattern among older individuals at high CVD risk

**DOI:** 10.1186/s12933-022-01659-6

**Published:** 2022-10-26

**Authors:** Jesus F. Garcia‑Gavilan, Margery A. Connelly, Nancy Babio, Christos S. Mantzoros, Emilio Ros, Jordi Salas‑Salvado

**Affiliations:** 1 Universitat Rovira i Virgili, Departament de Bioquimica i Biotecnologia, Unitat de Nutricio Humana, 43201 Reus, Tarragona, Spain; 2 Institut d’Investigacio Sanitaria Pere Virgili (IISPV), Reus, Spain; 3 Consorcio CIBER, Fisiopatologia de la Obesidad y Nutricion (CIBERObn), Instituto de Salud Carlos III (ISCIII), Madrid, Spain; 4 Laboratory Corporation of America® Holdings (Labcorp), Morrisville, Raleigh, NC USA; 5 Department of Medicine, Beth Israel Deaconess Medical Center, Harvard Medical School, Boston, MA 02215 USA; 6 Section of Endocrinology, VA Boston Healthcare System, Jamaica Plain, Boston, MA 02130 USA; 7 Lipid Clinic, Department of Endocrinology and Nutrition, Agust Pi i Sunyer Biomedical Research Institute (IDIBAPS), Hospital Clinic, University of Barcelona, Barcelona, Spain

## Correction: Cardiovascular Diabetology (2022) 21:189 https://doi.org/10.1186/s12933-022-01624-3

Following publication of the original article [1], the authors noticed the errors in the author group.

The typesetter has inadvertently missed to list Dr. Emilio Ros as the corresponding author of the article along with Dr. Jordi Salas-Salvadó. Also, the family name of the co-author “Christos S. Mantzoros” was incorrectly written as Christos S. Matzoros. Now, these changes have been corrected with this erratum.

The original article [1] has been updated.
