# Supplementary material for: Nut consumption is associated with a shift of the NMR lipoprotein subfraction profile to a less atherogenic pattern among older individuals at high CVD risk
Source: Cardiovasc Diabetol. 2022 Sep 20;21:189. doi: 10.1186/s12933-022-01624-3 (PMC9487141; doi:10.1186/s12933-022-01624-3)
Supplement: Supplementary file 1 — Additional file 1: Table S1. Lipoprotein particle parameters at baseline by tertiles of energy-adjusted nut consumption in a subcohort of the PREDIMED-Reus trial. Table S2. Apolipoproteins, small molecule metabolites, and markers of diabetes risk at baseline by tertiles of baseline energy-adjusted nut consumption at baseline in a subcohort of the PREDIMED-Reus trial. [file 12933_2022_1624_MOESM1_ESM.docx]

**Supplemental Table 1**. Lipoprotein particle parameters at baseline by tertiles of energy-adjusted nut consumption in a subcohort of the PREDIMED-Reus trial.

| **Variables** | **Tertile 1**  **(n = 65)** | **Tertile 3**  **(n = 65)** | ***ANCOVA***  ***P*-value** | **Tertile 1**  **(n = 65)** | **Tertile 3**  **(n = 65)** | ***ANCOVA***  ***P*-value** | **Tertile 1**  **(n = 65)** | **Tertile 3**  **(n = 65)** | ***ANCOVA***  ***P*-value** |
| --- | --- | --- | --- | --- | --- | --- | --- | --- | --- |
|  | **Total nuts** | | | **Walnuts** | | | **Non-walnut nuts** | | |
| **Food, g/d** | 3.1 (2.5, 3.7) | 29.7 (26.6, 32.8) |  | 0.8 (0.5, 1.2) | 14.4 (12.6, 16.2) |  | 1.2 (0.9, 1.5) | 17.1 (14.9, 19.3) |  |
| **Triglyceride-Rich Lipoprotein (VLDL) Particle Concentrations** | | | | | | | | | |
| **Total** | 0.05 (-0.15, 0.25) | 0.06 (-0.15, 0.27) | 0.994 | -0.03 (-0.22, 0.16) | 0.03 (-0.17, 0.23) | 0.722 | -0.09 (-0.27, 0.09) | 0.00 (-0.21, 0.21) | 0.586 |
| **Very large** | 0.22 (0.01, 0.43) | -0.10 (-0.30, 0.10) | 0.053 | **0.24 (0.03, 0.45)** | **-0.11 (-0.31, 0.09)†** | **0.035** | 0.11 (-0.10, 0.32) | -0.14 (-0.34, 0.06) | 0.131 |
| **Large** | 0.13 (-0.08, 0.34) | -0.09 (-0.28, 0.10) | 0.184 | 0.12 (-0.09, 0.33) | 0.05 (-0.14, 0.24) | 0.657 | 0.17 (-0.04, 0.38) | -0.11 (-0.31, 0.09) | 0.087 |
| **Medium** | 0.05 (-0.17, 0.27) | -0.04 (-0.23, 0.15) | 0.607 | 0.02 (-0.20, 0.24) | -0.04 (-0.24, 0.16) | 0.721 | 0.05 (-0.17, 0.27) | -0.11 (-0.31, 0.09) | 0.347 |
| **Small** | 0.02 (-0.18, 0.22) | -0.02 (-0.23, 0.19) | 0.836 | 0.05 (-0.15, 0.25) | -0.06 (-0.27, 0.15) | 0.506 | -0.09 (-0.29, 0.11) | 0.01 (-0.21, 0.23) | 0.535 |
| **Very Small** | 0.02 (-0.18, 0.22) | 0.09 (-0.10, 0.28) | 0.704 | -0.09 (-0.30, 0.12) | 0.09 (-0.12, 0.30) | 0.290 | -0.06 (-0.25, 0.13) | 0.02 (-0.18, 0.22) | 0.686 |
| **LDL Particle Concentrations** | | | | | | | | | |
| **Total** | 0.06 (-0.14, 0.26) | -0.07 (-0.26, 0.12) | 0.430 | **0.16 (-0.04, 0.36)** | **-0.20 (-0.41, 0.01)†** | **0.027** | -0.07 (-0.27, 0.13) | 0.03 (-0.15, 0.21) | 0.520 |
| **Large** | -0.11 (-0.33, 0.11) | 0.01 (-0.18, 0.20) | 0.460 | -0.01 (-0.22, 0.20) | -0.01 (-0.20, 0.18) | 0.993 | -0.04 (-0.27, 0.19) | 0.12 (-0.08, 0.32) | 0.294 |
| **Medium** | 0.10 (-0.12, 0.32) | -0.10 (-0.30, 0.10) | 0.259 | **0.19 (-0.03, 0.41)** | **-0.26 (-0.45, -0.07)†** | **0.011** | -0.06 (-0.28, 0.16) | 0.07 (-0.12, 0.26) | 0.474 |
| **Small** | 0.01 (-0.19, 0.21) | 0.01 (-0.19, 0.21) | 0.995 | -0.02 (-0.23, 0.19) | 0.03 (-0.16, 0.22) | 0.756 | -0.06 (-0.28, 0.16) | -0.08 (-0.28, 0.12) | 0.897 |
| **HDL Particle Concentrations** | | | | | | | | | |
| **Total** | -0.16 (-0.39, 0.07) | 0.09 (-0.10, 0.28) | 0.133 | -0.05 (-0.26, 0.16) | -0.05 (-0.25, 0.15) | 0.986 | **-0.22 (-0.45, 0.01)** | **0.12 (-0.08, 0.32)** | **0.043** |
| **Large** | **-0.22 (-0.42, -0.02)** | **0.12 (-0.07, 0.31**)**†** | **0.036** | -0.16 (-0.37, 0.05) | 0.04 (-0.16, 0.24) | 0.235 | -0.15 (-0.35, 0.05) | 0.12 (-0.06, 0.30) | 0.090 |
| **Medium** | 0.04 (-0.17, 0.25) | -0.04 (-0.25, 0.17) | 0.601 | 0.11 (-0.10, 0.32) | 0.04 (-0.19, 0.27) | 0.687 | 0.01 (-0.20, 0.22) | -0.01 (-0.21, 0.19) | 0.907 |
| **Small** | -0.08 (-0.30, 0.14) | 0.05 (-0.14, 0.24) | 0.451 | -0.06 (-0.27, 0.15) | -0.12 (-0.33, 0.09) | 0.735 | -0.13 (-0.35, 0.09) | 0.07 (-0.12, 0.26) | 0.223 |
| **H7P** | -0.13 (-0.34, 0.08) | 0.09 (-0.10, 0.28) | 0.180 | -0.04 (-0.23, 0.15) | 0.05 (-0.15, 0.25) | 0.583 | -0.15 (-0.36, 0.06) | 0.08 (-0.11, 0.27) | 0.172 |
| **H6P** | -0.09 (-0.29, 0.11) | 0.1 (-0.10, 0.30) | 0.248 | 0.00 (-0.20, 0.20) | 0.12 (-0.08, 0.32) | 0.462 | -0.09 (-0.29, 0.11) | 0.20 (0.01, 0.39) | 0.078 |
| **H5P** | -0.21 (-0.40, -0.02) | 0.04 (-0.16, 0.24) | 0.138 | -0.22 (-0.42, -0.02) | -0.06 (-0.26, 0.14) | 0.329 | -0.05 (-0.22, 0.12) | -0.01 (-0.21, 0.19) | 0.791 |
| **H4P** | -0.01 (-0.21, 0.19) | -0.01 (-0.22, 0.20) | 0.985 | 0.05 (-0.15, 0.25) | -0.08 (-0.29, 0.13) | 0.396 | 0.00 (-0.19, 0.19) | 0.01 (-0.20, 0.22) | 0.931 |
| **H3P** | 0.09 (-0.11, 0.29) | 0.01 (-0.19, 0.19) | 0.583 | 0.12 (-0.09, 0.33) | 0.15 (-0.05, 0.35) | 0.885 | 0.08 (-0.12, 0.28) | 0.02 (-0.17, 0.21) | 0.737 |
| **H2P** | -0.07 (-0.27, 0.13) | -0.01 (-0.20, 0.18) | 0.694 | 0.02 (-0.16, 0.20) | -0.05 (-0.26, 0.16) | 0.696 | -0.13 (-0.33, 0.07) | 0.05 (-0.14, 0.24) | 0.280 |
| **H1P** | -0.07 (-0.29, 0.15) | 0.12 (-0.07, 0.31) | 0.261 | -0.08 (-0.27, 0.11) | -0.06 (-0.25, 0.13) | 0.896 | -0.08 (-0.29, 0.13) | 0.07 (-0.12, 0.26) | 0.351 |
| **Mean Lipoprotein Particle size** | | | | | | | | | |
| **VLDL** | 0.18 (-0.02, 0.38) | -0.11 (-0.32, 0.10) | 0.096 | 0.13 (-0.08, 0.34) | 0.01 (-0.20, 0.22) | 0.475 | **0.19 (-0.01, 0.39)** | **-0.18 (-0.40, 0.04)†** | **0.026** |
| **LDL** | -0.06 (-0.29, 0.17) | -0.02 (-0.21, 0.17) | 0.803 | -0.04 (-0.26, 0.18) | 0.00 (-0.18, 0.18) | 0.832 | 0.00 (-0.24, 0.24) | 0.12 (-0.07, 0.31) | 0.479 |
| **HDL** | -0.17 (-0.39, 0.05) | 0.11 (-0.08, 0.30) | 0.079 | -0.08 (-0.27, 0.11) | 0.06 (-0.15, 0.27) | 0.370 | -0.15 (-0.37, 0.07) | 0.11 (-0.07, 0.29) | 0.110 |
| **Derived Triglyceride and Cholesterol Concentrations** | | | | | | | | | |
| **TG** | 0.19 (-0.05, 0.43) | -0.09 (-0.26, 0.08) | 0.094 | 0.16 (-0.07, 0.39) | -0.04 (-0.22, 0.14) | 0.224 | 0.12 (-0.11, 0.35) | -0.16 (-0.35, 0.03) | 0.100 |
| **TC** | -0.03 (-0.24, 0.18) | -0.02 (-0.21, 0.17) | 0.944 | 0.09 (-0.12, 0.30) | -0.17 (-0.36, 0.02) | 0.101 | -0.16 (-0.37, 0.05) | 0.09 (-0.09, 0.27) | 0.115 |
| **VLDL-TG** | 0.21 (-0.02, 0.44) | -0.10 (-0.27, 0.07) | 0.070 | 0.15 (-0.08, 0.38) | -0.04 (-0.22, 0.14) | 0.261 | 0.15 (-0.08, 0.38) | -0.17 (-0.36, 0.02) | 0.054 |
| **VLDL-C** | 0.12 (-0.09, 0.33) | -0.02 (-0.21, 0.17) | 0.391 | 0.06 (-0.15, 0.27) | -0.02 (-0.20, 0.16) | 0.640 | 0.02 (-0.18, 0.22) | -0.06 (-0.26, 0.14) | 0.629 |
| **LDL-C** | 0.01 (-0.19, 0.21) | -0.07 (-0.26, 0.12) | 0.608 | **0.14 (-0.07, 0.35)** | **-0.20 (-0.40, 0.00)†** | **0.035** | -0.10 (-0.29, 0.09) | 0.09 (-0.10, 0.28) | 0.239 |
| **HDL-C** | **-0.22 (-0.43, -0.01)** | **0.11 (-0.07, 0.29)†** | **0.036** | -0.10 (-0.30, 0.10) | 0.02 (-0.18, 0.22) | 0.481 | **-0.21 (-0.42, 0.00)** | **0.11 (-0.07, 0.29)†** | **0.040** |
| Lipid data are means (95% CI) of normalized values scaled in multiples of 1 SD with Blom’s rank-based inverse normal transformation data. Nuts groups were adjusted by energy intake and values are means (95% CI). *P*-values were obtained by ANCOVA adjusted by age, gender, body mass index (kg/m^2^), smoking status (ever smoker/never smoker), physical activity (met/day), diabetes (yes/no), dyslipidemia (yes/no), hypertension (yes/no), and statin treatment (yes/no).  Abbreviations: LDL, low-density lipoprotein; HDL, high-density lipoprotein; TRL-P, Triglyceride-Rich Lipoprotein Particle; TG, triglyceride; TC, total cholesterol; TRL, Triglyceride-Rich Lipoprotein; VLDL, very low-density lipoprotein.  † *P*-value <0.05 for comparison between T1 and T3 after the Tukey test. | | | | | | | | | |

**Supplemental Table 2**. Apolipoproteins, small molecule metabolites, and markers of diabetes risk at baseline by tertiles of baseline energy-adjusted nut consumption at baseline in a subcohort of the PREDIMED-Reus trial.

| **Variables** | **Tertile 1**  **(n = 65)** | **Tertile 3**  **(n = 65)** | ***ANCOVA***  ***P*-value** | **Tertile 1**  **(n = 65)** | **Tertile 3**  **(n = 65)** | ***ANCOVA***  ***P*-value** | **Tertile 1**  **(n = 65)** | **Tertile 3**  **(n = 65)** | ***ANCOVA***  ***P*-value** |
| --- | --- | --- | --- | --- | --- | --- | --- | --- | --- |
|  | **Total nuts** | | | **Walnuts** | | | **Nuts without walnuts** | | |
| **Food, g/d** | 3.1 (2.5, 3.7) | 29.7 (26.6, 32.8) |  | 0.8 (0.5, 1.2) | 14.4 (12.6, 16.2) |  | 1.2 (0.9, 1.5) | 17.1 (14.9, 19.3) |  |
| **Apolipoprotein Concentrations** | | | | | | | | | |
| **Apo B** | 0.04 (-0.16, 0.24) | -0.06 (-0.25, 0.13) | 0.538 | 0.12 (-0.08, 0.32) | -0.18 (-0.38, 0.02) | 0.055 | -0.10 (-0.30. 0.10) | 0.05 (-0.13. 0.23) | 0.368 |
| **Apo A-1** | -0.21 (-0.43, 0.01) | 0.08 (-0.10, 0.26) | 0.066 | -0.06 (-0.27, 0.15) | 0.01 (-0.18, 0.20) | 0.688 | **-0.23 (-0.46. 0.00)** | **0.12 (-0.06. 0.30)†** | **0.029** |
| **Amino Acid Concentrations** | | | | | | | | | |
| **BCAA** | **0.24 (0.04, 0.44)** | **-0.15 (-0.37, 0.07)†** | **0.012** | 0.24 (0.05, 0.43) | -0.05 (-0.25, 0.15)**†** | 0.058 | **0.21 (0.00. 0.42)** | **-0.15 (-0.34. 0.04)†** | **0.020** |
| **Valine** | **0.20 (0.00, 0.40)** | **-0.12 (-0.33, 0.09)†** | **0.045** | 0.22 (0.03, 0.41) | -0.08 (-0.28, 0.12)**†** | 0.057 | 0.11 (-0.11. 0.33) | -0.10 (-0.29. 0.09) | 0.191 |
| **Leucine** | **0.24 (0.04, 0.44)** | **-0.14 (-0.36, 0.08)†** | **0.017** | 0.20 (0.01, 0.39) | -0.07 (-0.29, 0.15) | 0.102 | **0.23 (0.03. 0.43)** | **-0.20 (-0.40. 0.00)*** | **0.008** |
| **Isoleucine** | 0.19 (-0.02, 0.40) | -0.08 (-0.29, 0.13) | 0.080 | 0.16 (-0.04, 0.36) | 0.08 (-0.10, 0.26) | 0.615 | **0.31 (0.10. 0.52)** | **-0.11 (-0.29. 0.07)*** | **0.007** |
| **Alanine** | 0.08 (-0.13, 0.29) | 0.01 (-0.19, 0.21) | 0.678 | 0.07 (-0.13, 0.27) | -0.03 (-0.21, 0.15) | 0.545 | 0.08 (-0.13. 0.29) | -0.02 (-0.22. 0.18) | 0.567 |
| **Glycine** | -0.04 (-0.26, 0.18) | 0.02 (-0.21, 0.25) | 0.737 | 0.11 (-0.12, 0.34) | -0.01 (-0.20, 0.18) | 0.426 | -0.06 (-0.28. 0.16) | 0.10 (-0.12. 0.32) | 0.326 |
| **Small Molecule Metabolites** | | | | | | | | | |
| **Glucose** | 0.18 (-0.01, 0.37) | -0.14 (-0.36, 0.08) | 0.060 | 0.07 (-0.13, 0.27) | -0.12 (-0.32, 0.08) | 0.267 | 0.14 (-0.05. 0.33) | -0.10 (-0.33, 0.13) | 0.167 |
| **Citrate** | -0.01 (-0.20, 0.18) | -0.03 (-0.25, 0.19) | 0.921 | -0.02 (-0.22, 0.18) | 0.10 (-0.12, 0.32) | 0.473 | 0.07 (-0.11. 0.25) | -0.03 (-0.25, 0.19) | 0.539 |
| **Ketone Body Concentrations** | | | | | | | | | |
| **Total KB** | 0.02 (-0.17, 0.21) | -0.03 (-0.24, 0.18) | 0.777 | 0.01 (-0.18, 0.20) | -0.10 (-0.30, 0.10) | 0.527 | -0.03 (-0.22. 0.16) | -0.01 (-0.22, 0.20) | 0.911 |
| **Beta-hydroxy-butyrate** | 0.01 (-0.19, 0.21) | -0.04 (-0.25, 0.17) | 0.809 | 0.00 (-0.20, 0.20) | -0.11 (-0.32, 0.10) | 0.530 | -0.04 (-0.25. 0.17) | 0.01 (-0.19, 0.21) | 0.770 |
| **Aceto-acetate** | 0.00 (-0.18, 0.18) | -0.01 (-0.22, 0.20) | 0.954 | 0.02 (-0.19, 0.23) | -0.06 (-0.25, 0.13) | 0.671 | -0.07 (-0.27. 0.13) | 0.03 (-0.18, 0.24) | 0.598 |
| **Acetone** | 0.05 (-0.14, 0.24) | -0.04 (-0.24, 0.16) | 0.589 | -0.03 (-0.21, 0.15) | -0.04 (-0.25, 0.17) | 0.958 | 0.05 (-0.14. 0.24) | -0.19 (-0.40, 0.02) | 0.157 |
| **Diabetes Risk Multimarkers** | | | | | | | | | |
| **LP-IR** | **0.24 (0.03, 0.45)** | **-0.12 (-0.31, 0.07)†** | **0.031** | 0.12 (-0.09, 0.33) | 0.02 (-0.19, 0.23) | 0.546 | **0.23 (0.02. 0.44)** | **-0.15 (-0.34, 0.04)†** | **0.018** |
| **DRI** | **0.20 (-0.02, 0.42)** | **-0.20 (-0.40, 0.00)†** | **0.020** | 0.10 (-0.10, 0.30) | 0.10 (-0.11, 0.31) | 0.984 | 0.25 (0.03. 0.47) | -0.06 (-0.25, 0.13) | 0.075 |
| **GlycA** | **0.10 (-0.10, 0.30)** | **-0.16 (-0.37, 0.05)*** | **<0.001** | **0.07 (-0.14, 0.28)** | **-0.06 (-0.28, 0.16)*** | **<0.001** | **0.07 (-0.14. 0.28)** | **-0.12 (-0.33, 0.09)*** | **<0.001** |
| **TMAO** | -0.09 (-0.31, 0.13) | -0.03 (-0.27, 0.21) | 0.759 | -0.14 (-0.35, 0.07) | -0.18 (-0.39, 0.03) | 0.797 | -0.02 (-0.24. 0.20) | 0.01 (-0.22, 0.24) | 0.864 |
| Metabolomics data are means (95% CI) of normalized values scaled in multiples of 1 SD with Blom’s rank-based inverse normal transformation data. Nuts groups were adjusted by energy intake and values are means (95% CI). *P*-values were obtained by ANCOVA adjusted by age, gender, body mass index (kg/m^2^), smoking status (ever smoker/never smoker), physical activity (met/day), diabetes (yes/no), dyslipidemia (yes/no), hypertension (yes/no), and statin treatment (yes/no).  Abbreviations: Apo, apolipoprotein; BCAA, Branched-Chain Amino Acids; KB, ketone body; LP-IR, lipoprotein insulin resistance; DRI, diabetes risk index; TMAO, Trimethylamine N-oxide.  † *P*-value <0.05 for comparison between T1 and T3; * *P*-value <0.01 for comparison between T1 and T3 after the Tukey test. | | | | | | | | | |
